# Novel Insights into Selected Disease-Causing Mutations within the *SLC35A1* Gene Encoding the CMP-Sialic Acid Transporter

**DOI:** 10.3390/ijms22010304

**Published:** 2020-12-30

**Authors:** Bożena Szulc, Yelyzaveta Zadorozhna, Mariusz Olczak, Wojciech Wiertelak, Dorota Maszczak-Seneczko

**Affiliations:** Faculty of Biotechnology, University of Wroclaw, 14A F. Joliot-Curie St., 50-383 Wroclaw, Poland; bozena.szulc@uwr.edu.pl (B.S.); zadorozhna.yelyzaveta@gmail.com (Y.Z.); mariusz.olczak@uwr.edu.pl (M.O.); wojciech.wiertelak@uwr.edu.pl (W.W.)

**Keywords:** CMP-sialic acid transporter, sialylation, congenital disorder of glycosylation, mutation, Golgi apparatus, lectin, N-glycan, O-glycan, glycolipid, protein dimerization

## Abstract

Congenital disorders of glycosylation (CDG) are a group of rare genetic and metabolic diseases caused by alterations in glycosylation pathways. Five patients bearing CDG-causing mutations in the *SLC35A1* gene encoding the CMP-sialic acid transporter (CST) have been reported to date. In this study we examined how specific mutations in the *SLC35A1* gene affect the protein’s properties in two previously described SLC35A1-CDG cases: one caused by a substitution (Q101H) and another involving a compound heterozygous mutation (T156R/E196K). The effects of single mutations and the combination of T156R and E196K mutations on the CST’s functionality was examined separately in CST-deficient HEK293T cells. As shown by microscopic studies, none of the CDG-causing mutations affected the protein’s proper localization in the Golgi apparatus. Cellular glycophenotypes were characterized using lectins, structural assignment of N- and O-glycans and analysis of glycolipids. Single Q101H, T156R and E196K mutants were able to partially restore sialylation in CST-deficient cells, and the deleterious effect of a single T156R or E196K mutation on the CST functionality was strongly enhanced upon their combination. We also revealed differences in the ability of CST variants to form dimers. The results of this study improve our understanding of the molecular background of SLC35A1-CDG cases.

## 1. Introduction

Cells present a remarkable diversity of glycan structures, all of which play important roles in mediating essential intra- and intercellular processes. Although vertebrate glycans vary greatly in structure, composition and function, their common feature is the presence of sialic acid as the terminal sugar. Sialic acids comprise a group of charged 9-carbon backbone sugars, of which 5-*N*-acetylneuraminic acid (Neu5Ac) is the most abundant in humans. These sugars occupy terminal positions of N- and O-glycans, glycosphingolipids and some GPI anchors and constitute essential components of surface glycoconjugates and secreted proteins. Their terminal positions in glycans define the functions these sugars fulfill in the cell. Sialic acids mediate cell-to-cell interactions during the immune response, and among many other roles, ensure proper functioning of cardiovascular and nervous systems [1]. One of the earliest examples of their action is the control of the alternative complement system through the interaction of surface sialic acid with factor H [2]. Many sialic-acid-binding proteins, notably selectins and siglecs, regulate a wide range of biological phenomena [3]. L, E and P-selectins mediate interactions between leukocytes, endothelial cells and platelets [4,5,6]. Among siglecs, MAG was shown to be involved in neuron–glial cell interactions [7,8], while other proteins of this family could be essential for immune cells, which they are widely expressed on [9]. As an essential component of brain gangliosides, sialic acid helps them fulfill their functions in regulating the activity of various membrane receptors and enables cell-to-cell recognition [10]. Additionally, sialic acid-rich glycans give specific properties to the cells they occupy. A prominent example is the polysialylation of the neural cell adhesion molecule (NCAM), which ensures proper trafficking of neural progenitors during brain development [11,12]. Such a diverse repertoire of functions makes sialic acids a particularly important feature of cellular glycans.

The biosynthesis of the majority of cellular glycans occurs in a stepwise manner in the lumen of the endoplasmic reticulum (ER) and Golgi apparatus. As the nascent glycan passes through these compartments, specific monosaccharide residues are added by respective glycosyltransferases from activated sugar donors. Nucleotide sugars are synthesized and activated in the cytoplasm, while CMP-sialic acid activation occurs in the nucleus; therefore, they have to be delivered into respective compartments in order to serve as sugar donors. This role is played by nucleotide sugar transporters (NSTs), a family of solute carriers localized in the membranes of the ER and/or Golgi apparatus. By acting as antiporters, they transport nucleotide sugars into the ER or Golgi lumen in exchange for respective nucleoside monophosphates [13,14]. The SLC35 family, which NSTs belong to, is represented by a large number of proteins with a putative transporting activity. Many of them have been experimentally assigned with single or even multiple sugar specificities, some have been suggested to have regulatory roles in glycosylation and beyond [15,16,17], and the functions of others are yet to be determined [18]. Nucleotide sugar transporters are capable of assembling into homomeric complexes [18,19,20,21,22,23,24,25] and forming heteromeric assemblies with other NSTs and glycosyltransferases [23,25,26,27,28]. The presence of multimeric transporter-transferase complexes in living cells was proposed to serve as a mechanism to facilitate glycosylation reactions by drawing closer NSTs and transferases which share the same nucleotide sugar substrates [27]. Although the tendency of glycosyltransferases to associate into complex structures has been known for decades [29], the spatial organization of other proteins involved in glycan biosynthesis remains largely unexplored.

The first studies on the CMP-sialic acid transporter (CST; SLC35A1) became possible with the isolation of CHO glycosylation mutants based on their resistance to wheat germ agglutinin (WGA). Two mutant cell lines, Lec2 [30] and clone 1021 [31], were generated independently and both showed a drastic reduction in sialylation, caused by a CMP-sialic acid transport defect [32]. This was further confirmed via complementation cloning of murine [33] and hamster [34] CSTs and their overexpression in mutant cells of the Lec2 complementation group. Both proteins upon overexpression were able to reverse the Lec2 phenotype of CHO mutants and localized to the Golgi apparatus. Furthermore, *Saccharomyces cerevisiae* expressing a murine CST gained the ability to transport CMP-sialic acid, confirming the protein’s predicted function in the cell [35]. The human CST was identified shortly after. Similarly to its murine and hamster homologs, it was able to correct the Lec2 phenotype when overexpressed, and the microsomal vesicles isolated from these cells exhibited high CMP-sialic acid transport activity [36]. In addition to the well-established Lec2 glycosylation mutants, a new CHO derivative line, MAR-11 (also known as CHO-gmt1), has been developed more recently. These cells were isolated based on their resistance to *Maackia amurensis* agglutinin (MAA) and contain a point mutation in the *SLC35A1* gene, resulting in a premature stop codon [37]. It is worth noting that glycosylation mutants surviving lectin treatment could potentially obtain additional off-target mutations, which raises the need to create cell lines harboring specific modifications using targeted genome editing.

Structural data on the CMP-sialic acid transporter remained limited for a long time. Epitope insertion experiments revealed that murine CST contains 10 transmembrane helices, with both N- and C-termini facing the cytosol [38]. Further studies using human UDP-galactose transporter (UGT) and CMP-sialic acid transporter chimeras demonstrated that for human UGT, helices 1 and 8 were essential for substrate recognition, while N- and C-terminal cytoplasmic regions were dispensable [39,40]. Importantly, the introduction of CST transmembrane helices 2, 3 and 7 into UGT led to a dual sugar specificity of the resulting transporter, indicating that some NSTs could potentially have multiple substrate-binding sites [40]. The importance of these transmembrane helices in substrate recognition was further confirmed in a crystal structure of the CST from *Zea mays* [41]. The recently resolved crystal structures of mouse and maize CSTs provided many insights into the mode of action of the transporter. Both transporters exhibit high substrate specificity; CMP mainly contributes to substrate recognition—sialic acid plays a smaller role [41,42]. It has been proposed that the transporter undergoes conformational changes from a fully open to a partially occluded state, where the bound substrate is shielded with transmembrane domains 1 and 8. Meanwhile, transmembrane domains 5, 9 and 10 were proposed to be involved in transport [42]. Although available structural data strongly support the antiport mechanism of action, the maize CST was shown to function as a uniporter as well, albeit less efficiently. Both mouse and maize CSTs were reported to form homodimers in crystals; however, so far there has not been any evidence on whether these interactions occur in the Golgi membranes of living cells and are relevant in vivo [41,42]. Additionally, recent studies on the yeast GDP-mannose (GDP-Man) transporter, Vrg4, showed that the protein requires a short-chain lipid environment for maximum activity and therefore suggested that the transport of GDP-Man is only efficient in the Golgi membranes, where the lipid bilayer is appropriately thin [23,43]. This observation could also hold true for the mammalian nucleotide sugar transporters.

Having one of the key roles in sialylation, the CMP-sialic acid transporter can regulate many physiological processes—directly and indirectly. Due to its terminal position in the glycan structures, sialic acid, and consequently CST, often mediate attachment and internalization of a wide range of viruses, including polyomaviruses, coronaviruses, some rotaviruses, reoviruses [44,45], influenza virus [46] and others. Modulation of the CST expression has been shown to either prevent viral entry and replication [45,46,47] or facilitate it, as is the case for vesicular stomatitis virus (VSV) and mutant PML-associated JC polyomaviruses [48,49]. Additionally, the CST has been proposed to play a role in mediating a pro-apoptotic response during VSV infections [48].

Mutations in the *SLC35A1* gene, encoding the CMP-sialic acid transporter, are known to cause congenital disorders of glycosylation (CDG). These rare genetic and metabolic disorders arise from defects in glycosylation pathways and usually result in improper glycan assembly. The first report of SLC35A1-CDG involved a patient with two microdeletions in allele 1, resulting in a premature stop codon and a 130-nucleotide deletion in allele 2, which likely caused alterations in splicing and synthesis of a truncated protein. In-depth analysis of the protein functionality was not possible, since truncated versions of CST were rapidly degraded [50]. Another known case of SLC35A1-CDG was caused by a point mutation, Q101H, which led to a reduction of the transporter’s activity by approximately 50% [51]. Later, upon the identification of the structure of the murine CST, it was postulated that the Q101H mutation might affect the protein’s interaction with the substrate [42]. Interestingly, this CDG-causing mutation has also been shown to affect the *O*-mannosyl glycan synthesis on alpha-dystroglycan (α-DG) proteins [52]. Reduced *O*-mannosylation on α-DG could be restored after ribitol supplementation, suggesting a limited CDP-ribitol supply in cells with defective *SLC35A1* genes and an indirect role of CST in CDP-ribitol transport [53]. Another reported SLC35A1-CDG patient exhibited a 9-fold reduction in CMP-sialic acid transport. The sialylation defect was caused by a compound heterozygous mutation, T156R/E196K, which might impede the conformational changes during transport [54]. Specifically, the T156R mutation has been suggested to impair the protein’s ability to dimerize [41]. Both the latter patient and the one bearing Q101H mutation displayed neurological symptoms, including intellectual disability, hypotonia, ataxia and seizures [51,53]. The most recently reported SLC35A1-CDG case describes two patients with a homozygous S147P mutation, who, similarly to others affected by this disease [50,51], experienced macrothrombocytopenia. The condition was caused by hyposialylation of blood platelets and their increased clearance [55]. The essential roles of the CMP-sialic acid transporter in platelet generation and clearance have been further emphasized in a study on a mouse model with SLC35A1-deficient megakaryocytes and platelets [56]. Taken together, earlier and recent studies on CDG broaden our understanding of the CST structure and physiological importance.

In this study we developed a novel human cell-based model enabling functional studies on the CMP-sialic acid transporter and SLC35A1-CDG by inactivating the *SLC35A1* gene in HEK293T cells. Next, we used the CST-deficient cells for stable expression of epitope-tagged wild-type and selected disease-causing variants of the CST protein (Q101H, T156R, E196K and a double mutant consisting of the two latter mutations), resulting in a panel of seven cell lines in total. Although the T156R and E196K mutations have not occurred within the same protein in any patient’s cells, we decided to combine them and compare the behavior of such an artificial CST variant with the performances of the variants bearing the corresponding single mutations. Analysis of subcellular localization of the CST variants revealed that none of the mutations affected the proper Golgi localization of the CST. Glycophenotypes of the resulting cell lines were subsequently characterized using lectins, structural assignment of N- and O-glycans and analysis of glycolipids. Inactivation of the *SLC35A1* gene did not result in a complete disappearance of sialylated N-glycans. In contrast, sialylated O-glycans and glycolipids were more severely affected by the CST loss. Disease-causing CST variants were able to restore sialylation to a varying extent which depended on the class of glycoconjugates. However, sialylation of all classes of glycoconjugates we have analyzed was least efficiently restored by the expression of the double T156R/E196K mutant. Finally, we examined the ability of wild-type and disease-causing CST variants to form dimers in living cells using a split luciferase complementation assay. We revealed that the wild-type human CST dimerized in its native environment. The T156R variant and double T156R/E196K mutant also displayed similar tendencies. In contrast, the potential of Q101H and E196K variants to form dimers appeared to be disabled, as they both failed to reconstitute a functional luciferase to a significant extent. Our results extend the knowledge on the etiology of SLC35A1-CDG and demonstrate that the human CMP-sialic acid transporter is able to form dimers in the Golgi membranes of living cells.

## 2. Results

### 2.1. Inactivation of the SLC35A1 Gene in HEK293T Cells via CRISPR/Cas9 Strategy

**Figure 1:**
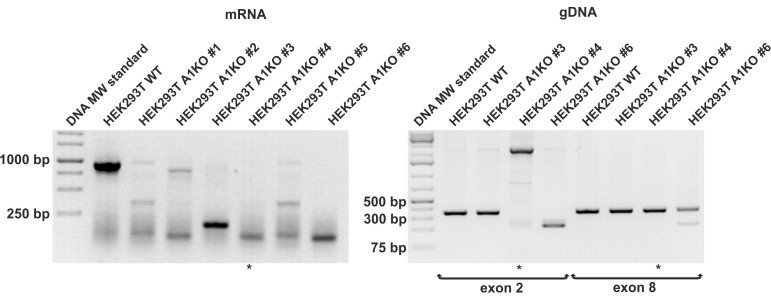
Verification of a knock-out of the *SLC35A1* gene in HEK293T cells. Genomic DNA (gDNA) and total RNA were isolated from the wild-type HEK293T cells and several stable transfectants, and either PCR (DNA) or RT-PCR (mRNA) was performed using *SLC35A1* gene-specific primers. The amplified products were separated in 1.3% (*w*/*v*, mRNA) and 1.8% (*w*/*v*, gDNA) agarose gels and visualized with ethidium bromide. The clone selected for further use is indicated with an asterisk (*). A1KO, cells with an inactive *SLC35A1* gene.

To inactivate the *SLC35A1* gene in HEK293T cells, we employed the CRISPR/Cas9 strategy. For this purpose, the cells were transfected with the appropriate plasmids and RNA, and several independent clones were isolated. To confirm that the gene inactivation was effective, genomic DNA and total RNA were isolated from HEK293T wild-type and putative *SLC35A1* knock-out cells. First, clones 1–6 were analyzed by the means of RT-PCR using total RNA as a template and gene-specific primers designed to amplify a 927 bp product (Table S1). At this stage, clones 3, 4 and 6 were selected, as no products that could possibly correspond to an unaffected *SLC35A1* sequence were observed (Figure 1). In the next step, genomic DNA was analyzed using PCR. As the crRNAs used in this study target exon 2 (crRNAs 1 and 3) and exon 8 (crRNA 2), primers were designed to amplify 351 bp (exon 2) and 365 bp (exon 8) products (Table S2). The obtained results suggested that in clone 4 a large insertion within exon 2 had occurred, while mutations that had arose in clones 3 and 6 appeared less dramatic. We therefore concluded that the *SLC35A1* gene in clone 4 was successfully knocked-out.

### 2.2. Analysis of Subcellular Localization of CST Variants

**Figure 2:**
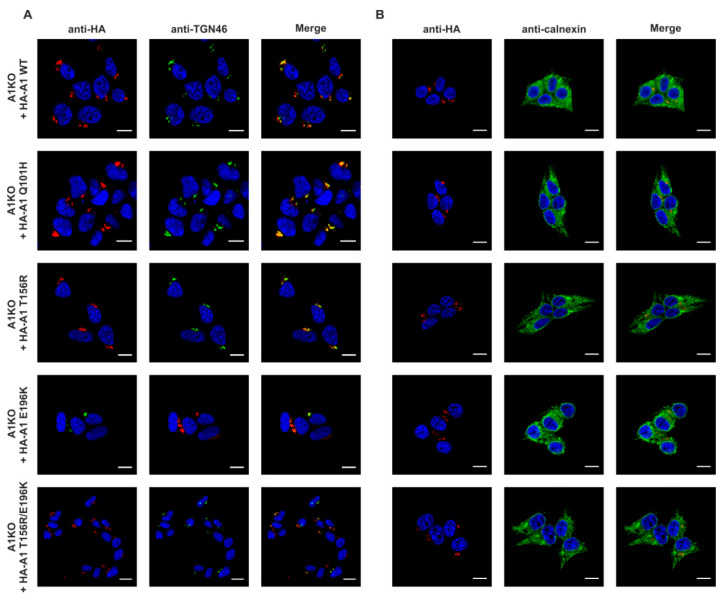
Analysis of subcellular localization of the wild-type and disease-causing CST variants. CST-deficient HEK293T cells overexpressing recombinant CST variants were subjected to indirect immunofluorescent staining with anti-HA (red) and anti-organelle marker (green) antibodies ((**A**), counterstaining of the Golgi apparatus; (**B**), counterstaining of the ER). Cell nuclei were counterstained with 4′,6-diamidino-2-phenylindole (DAPI). Scale bar = 10 μm. TGN46, trans-Golgi network integral membrane protein 2. A1, SLC35A1.

In the next step we stably expressed wild-type and disease-causing CST variants in the selected CST-deficient clone. Q101H, T156R and E196K CST variants were obtained via site-directed mutagenesis using primers listed in Table S3 (the mutated residues are shown in Figures S1 and S2, respectively). Protein expression in the stable transfectants was verified by Western blotting using HRP-conjugated anti-HA antibody (Figure S3A) and PCR carried out on cDNA samples (Figure S3B) using primers listed in Tables S1 and S4. These results confirmed expression of all CST variants and did not show any significant degradation of the recombinant proteins. As the mutations in the *SLC35A1* gene could potentially affect the subcellular localization of the resulting proteins, immunofluorescence staining was performed in order to identify whether the mutant CST variants localize to the Golgi apparatus or not. The recombinant, HA-tagged CST variants were detected using an epitope-specific antibody. The trans-Golgi network integral membrane protein 2 (TGN46) and calnexin were used as markers of the Golgi apparatus and ER, respectively. As shown in Figure 2, both wild-type and disease-causing variants of the CST were expressed by the knock-out cells, properly localized to the Golgi apparatus (panel A) and showed no co-localization with the ER marker calnexin (panel B). We therefore concluded that the mutations we analyzed do not affect subcellular localization of the resulting CST variants.

### 2.3. Analysis of Cell Surface Glycoconjugates Using Lectin-Based Flow Cytometry

**Figure 3:**
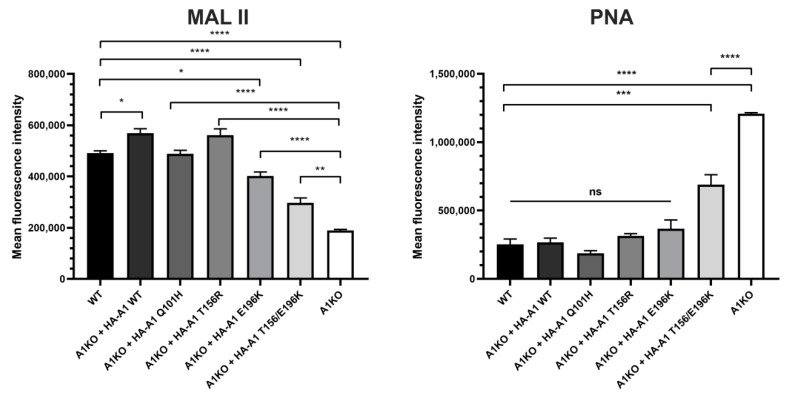
Analysis of reactivity of cell surface glycoconjugates with selected lectins using flow cytometry. PNA, *Arachis hypogaea* lectin; MAL II, *Maackia amurensis* lectin II. Data are presented as mean fluorescence intensity of three independent biological replicates, each performed for 10,000 cells ± SEM. For statistical analysis of the data, one-way ANOVA with Tukey’s post-hoc test was used. Knock-out cells overexpressing CST variants were compared with both wild-type and knock-out cells. Statistical significance was assigned top-value < 0.05. ns, not significant; *, *p* < 0.05; **, *p* < 0.01; ***, *p* < 0.001; ****, *p* < 0.0001.

For the initial evaluation of the glycophenotype resulting from inactivation of the *SLC35A1* gene in HEK293T cells and the ability of CST variants to restore sialylation, we labeled intact cells with the selected lectins and analyzed them using flow cytometry. This approach allowed us to selectively examine cell surfaces, and thus fully mature glycoconjugates. We selected two lectins: *Arachis hypogaea* lectin (PNA) and *Maackia amurensis* lectin II (MAL II). PNA is specific for Galβ1-3GalNAc structures typical for core 1 O-glycans, while MAL II recognizes sialic acid residues attached via α2–3 linkage to an underlying sugar. Upon inactivation of the *SLC35A1* gene, the reactivity of PNA with cell surface glycoconjugates should increase due to enhanced exposure of its target epitope resulting from a loss of terminal sialylation. Indeed, CST-deficient cells displayed significantly higher reactivity with this lectin. Expression of the wild-type CST and the Q101H, T156R and E196K single mutants restored reactivity of cell surface glycoconjugates with PNA to levels typical of the wild-type cells. However, cells expressing the double T156R/E196K mutant displayed reactivity with PNA at a level lower than CST-deficient cells, but at the same time, at a significantly higher level than the wild-type cells (Figure 3). Since MAL II binds to sialic acid, a decrease of its reactivity with cell surface glycoconjugates upon inactivation of the *SLC35A1* gene was expected. Indeed, reactivity of MAL II with cell surface glycoconjugates produced by the knock-out cells was significantly lower than that of the wild-type cells. Expression of the wild-type CST and Q101H and T156R variants restored sialylation to a level typical of the wild-type cells. In contrast, the E196K variant and the double T156R/E196K mutant were not able to restore sialylation to a similar extent, although the corresponding glycoconjugates bound significantly more MAL II than the knock-out cells (Figure 3). As shown by the results obtained using both lectins, the double T156R/E196K mutant turned out to be least capable of restoring the wild-type glycophenotype in the knock-out cells upon overexpression.

### 2.4. Analysis of Cellular Glycoproteins Using Lectin Blotting

**Figure 4:**
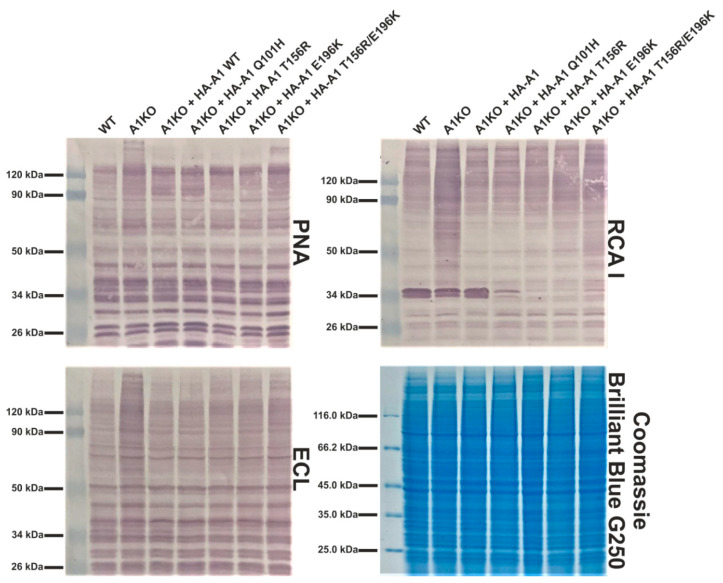
Analysis of cellular glycoproteins using lectin blotting. Proteins present in total cell lysates were separated by SDS-PAGE, transferred onto a nitrocellulose membrane and probed with selected biotinylated lectins followed by avidin D conjugated with alkaline phosphatase and colorimetric detection. SDS-PAGE-resolved proteins were stained with Coomassie Brilliant Blue G250 as a loading control. PNA, *Arachis hypogea* lectin; RCA I, *Ricinus communis* agglutinin I; ECL, *Erythrina cristagalli* lectin. A1, SLC35A1. The bands around 34 kDa strongly reactive towards RCA I observed in some lanes are most probably derived from the fetal bovine serum, in the presence of which the cells were cultured.

As an alternative method to preliminarily test how selected CDG-causing mutations in the *SLC35A1* gene affect the cells’ glycophenotype, lectin reactivity against cell lysate glycoproteins was examined. We selected the following lectins: PNA, whose specificity is described in the previous paragraph, *Ricinus communis* agglutinin I (RCA I), which is specific for Gal and GalNAc residues and *Erithrina cristagalli* lectin (ECL), which is specific for Galβ1-4GlcNAc-R structures. As shown in Figure 4, all these lectins displayed a higher reactivity towards glycoproteins synthesized by the CST-deficient cells when compared with the wild-type cells. These results suggested an exposure of the underlying sugars (Gal/GalNAc) in glycoproteins, most likely caused by a defect in sialic acid incorporation. However, there were subtle differences in lectin reactivity towards glycoproteins synthesized by the knock-out cells expressing CST variants. In the case of PNA, the Q101H, T156R and E196K variants restored sialylation to a level similar to the cells expressing the wild-type CST, while the double T156R/E196K mutant was slightly less efficient in restoring sialylation. In the case of RCA I and ECL, only the Q101H variant corrected sialylation to a similar extent as the wild-type protein, and again, the double mutant was the least capable of restoring sialylation.

### 2.5. Structural Analysis of N-Glycans

**Figure 5:**
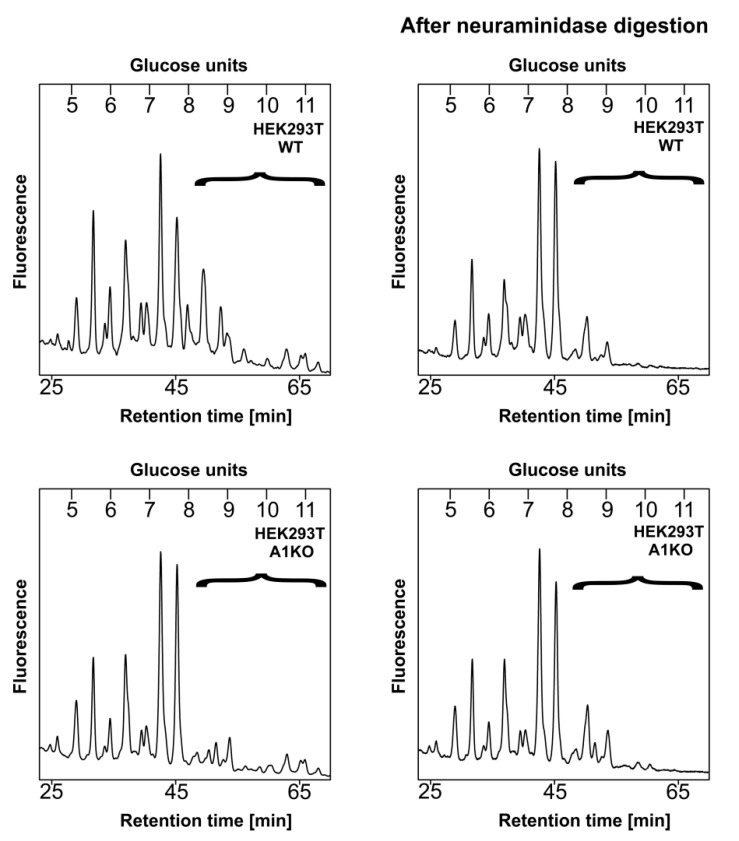
HPLC analysis of N-glycans released from the secreted reporter glycoprotein. N-Glycans derived from a modified SEAP overexpressed in wild-type (WT, top panel) and CST-deficient (A1KO, bottom panel) HEK293T cells were fluorescently labeled with 2-AB, purified and separated on the normal phase GlycoSep N column using HPLC. The left panel represents intact (undigested) structures, while the right panel corresponds to structures obtained upon a neuraminidase treatment. Respective glycans are expressed in glucose units. Sialylated structures are enclosed in brackets. Representative data from two independent analyses with a similar tendency are shown. A1, SLC35A1.

To obtain more details on the glycoprotein sialylation by the obtained cell lines, we employed structural analysis of enzymatically released, 2-AB-labeled N-glycans using two different strategies. The first approach involved separation of N-glycans released from an engineered secreted reporter glycoprotein, i.e., secreted alkaline phosphatase (SEAP) isolated from the conditioned media (data not published, manuscript in preparation) using high performance liquid chromatography (HPLC). N-Glycans derived from SEAP are expected to contain mature, fully processed structures including sialylated species; therefore, the possibility that sialylation is missing or underrepresented in a sample obtained from the wild-type cells is very low. This step was performed only for the wild-type and knock-out cells. The corresponding profiles are shown in Figure 5. The right panel represents HPLC profiles of the intact N-glycans released from SEAP. The left panel depicts profiles of N-glycans that had been digested with a neuraminidase prior to HPLC separation. This step resulted in the disappearance of the largest N-glycan species from both profiles, suggesting that in the CST-deficient cells some sialylation persists.

**Figure 6:**
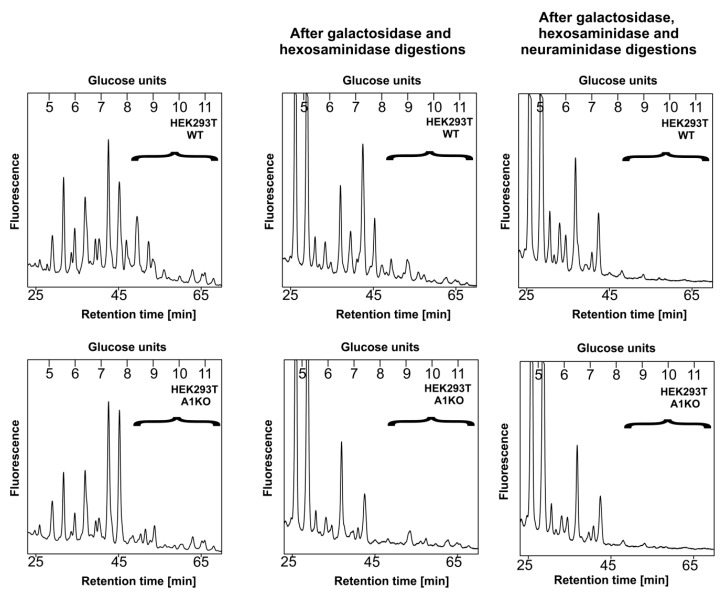
HPLC analysis of N-glycans released from the secreted reporter glycoprotein. N-glycans derived from a modified SEAP overexpressed in wild-type (WT, top panel) and CST-deficient (A1KO, bottom panel) HEK293T cells were fluorescently labeled with 2-AB, purified and separated on the normal phase GlycoSep N column using HPLC. The left panel represents intact (undigested) structures; the middle panel reflects structures obtained upon a combined digestion with galactosidase and hexosaminidase; and the right panel reflects structures obtained upon a digestion with neuraminidase followed by a combined digestion with galactosidase and hexosaminidase. Respective glycans are expressed in glucose units. Sialylated structures are enclosed in brackets. Representative data from two independent analyses with similar tendencies are shown. A1, SLC35A1.

In parallel, we compared the profiles of the undigested N-glycans with the ones obtained after combined digestion of the samples with a galactosidase and hexosaminidase. This approach should not cause any changes in sialylated N-glycans, as galactose residues capped with sialic acid would be resistant to such a treatment; therefore, any complex species that remain after such a treatment should be considered sialylated. Besides, elimination of structures terminating with galactose should make the resulting profile easier to analyze, as sialylated structures that remain should become more pronounced. After this step, the profile corresponding to the wild-type cells was much more complex compared to the knock-outs (Figure 6, middle panel), which suggested that the latter cells did not produce as many sialylated N-glycans as the former, as there were more oligosaccharides sensitive to galactosidase and hexosaminidase digestion present in the initial sample derived from the knock-outs compared to wild-type cells. However, when the digestion with these two enzymes was preceded with a neuraminidase treatment (Figure 6, right panel), more similar profiles were obtained for both cell lines, which most likely resulted from an increased subset of sialylated species in the sample derived from the wild-type cells within the indicated range (brackets) and not from, e.g., fucosylation or the presence of high mannose structures.

**Figure 7:**
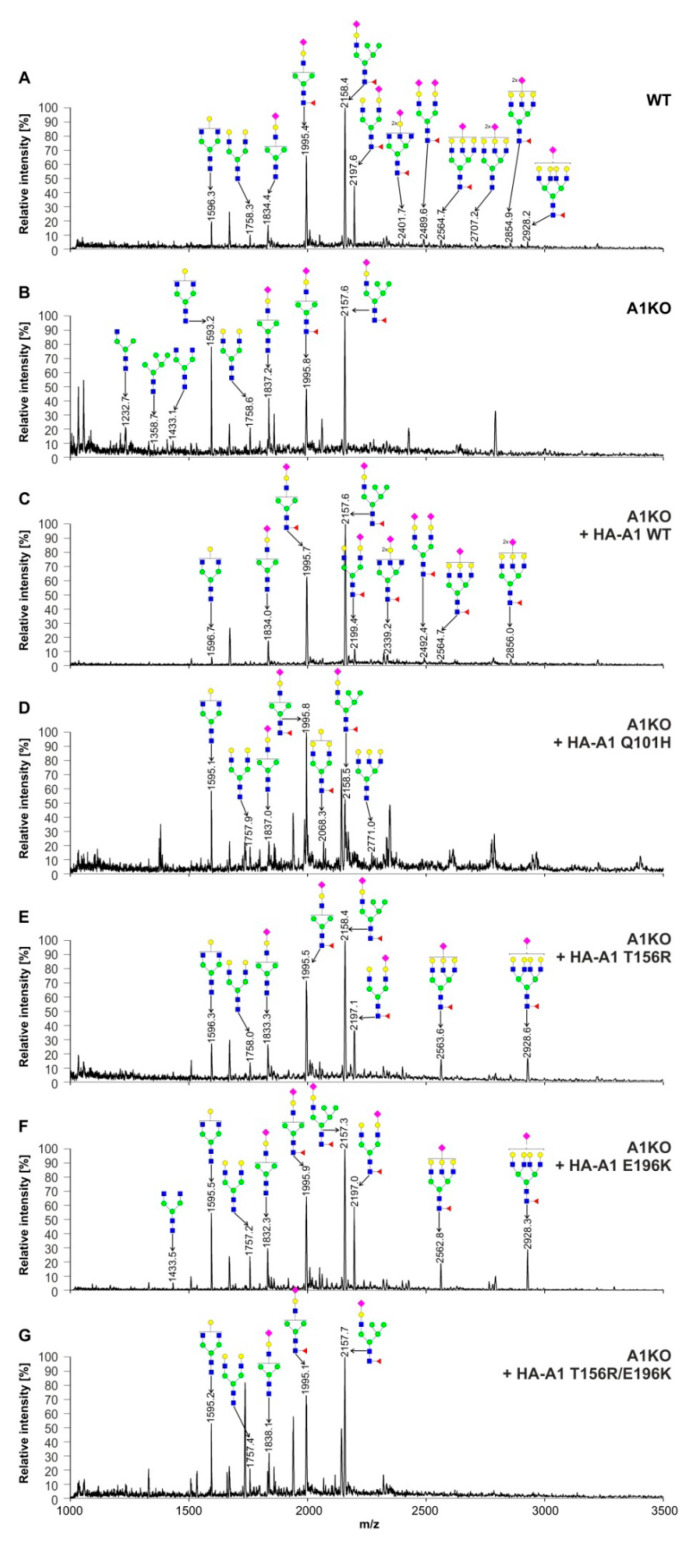
Structural analysis of N-glycans. MALDI-TOF MS characterization of 2-AB-labeled N-glycans released from cellular glycoproteins produced by the wild-type HEK293T cells (WT) (**A**), CST-deficient cells (**B**), CST-deficient cells expressing wild-type CST (**C**), CST-deficient cells expressing the Q101H CST variant (**D**), CST-deficient cells expressing the T156R CST variant (**E**), CST-deficient cells expressing the E196K CST variant (**F**) and CST-deficient cells expressing the T156R/E196K CST variant (**G**). N-Glycan composition was estimated using the GlycoWorkBench tool (2.1; EuroCarbDB). Identified peaks were labeled with mass information and cartoon representations of putative N-glycan chemical structures (based on biosynthetic knowledge). Green circles, mannose; blue squares, *N*-acetylglucosamine; yellow circles, galactose; red triangles, fucose; pink diamonds, sialic acid. A1, SLC35A1.

In order to evaluate the ability of individual CST variants to restore N-glycan sialylation, we analyzed 2-AB-labeled N-glycans released from cellular glycoproteins using matrix assisted laser desorption/ionization-time-of-flight mass spectrometry (MALDI-TOF MS) in negative-ion mode. The results of this approach are shown in Figure 7. Among N-glycans derived from the wild-type HEK293T cells, ten sialylated species were detected in total (Figure 7A). Inactivation of the *SLC35A1* gene resulted in a complete disappearance of seven sialylated structures, but three of them could still be detected (Figure 7B, m/z of ≈1837, ≈1996 and ≈2158), which is in line with the results of HPLC analysis. Interestingly, also species that are not direct precursors of sialylated structures (m/z of ≈1233, 1359 and 1433) appeared in this spectrum. Expression of the wild-type CST in the knock-out cells restored the majority of sialylated structures (Figure 7C). The T156R (Figure 7E) and E196R (Figure 7F) CST variants were also able to restore some sialylation, but expression of either the Q110H CST variant (Figure 7D) or the double T156R/E196K mutant (Figure 7F) did not improve N-glycan sialylation at all. It should be noted that even best-performing disease-causing CST variants were only able to restore monosialylated species and not those containing two sialic acid residues.

### 2.6. Structural Analysis of O-Glycans

To further examine the impacts of the CST variants on sialylation of glycoproteins, we employed the cellular O-glycome reporter/amplification (CORA) strategy developed by Kudelka et al. [57] to identify O-glycans produced by the wild-type and CST-deficient cells and the knock-out cells expressing different CST variants. This class of glycoconjugates has not been extensively explored in CST-deficient cell-based models and SLC35A1-CDG patients. HEK293T cells synthesize a wide range of sialylated O-glycan structures and are therefore a more suitable model for studying the impact of the CST on sialylation of O-glycans than CHO cells, which produce a limited number of sialylated O-linked oligosaccharides [58]. To analyze the O-glycome of the HEK293T cell line panel, cells were cultured in the presence of a modified acceptor O-glycan; the resulting oligosaccharides were isolated from the conditioned media, permethylated and analyzed using MALDI-TOF MS.

**Figure 8:**
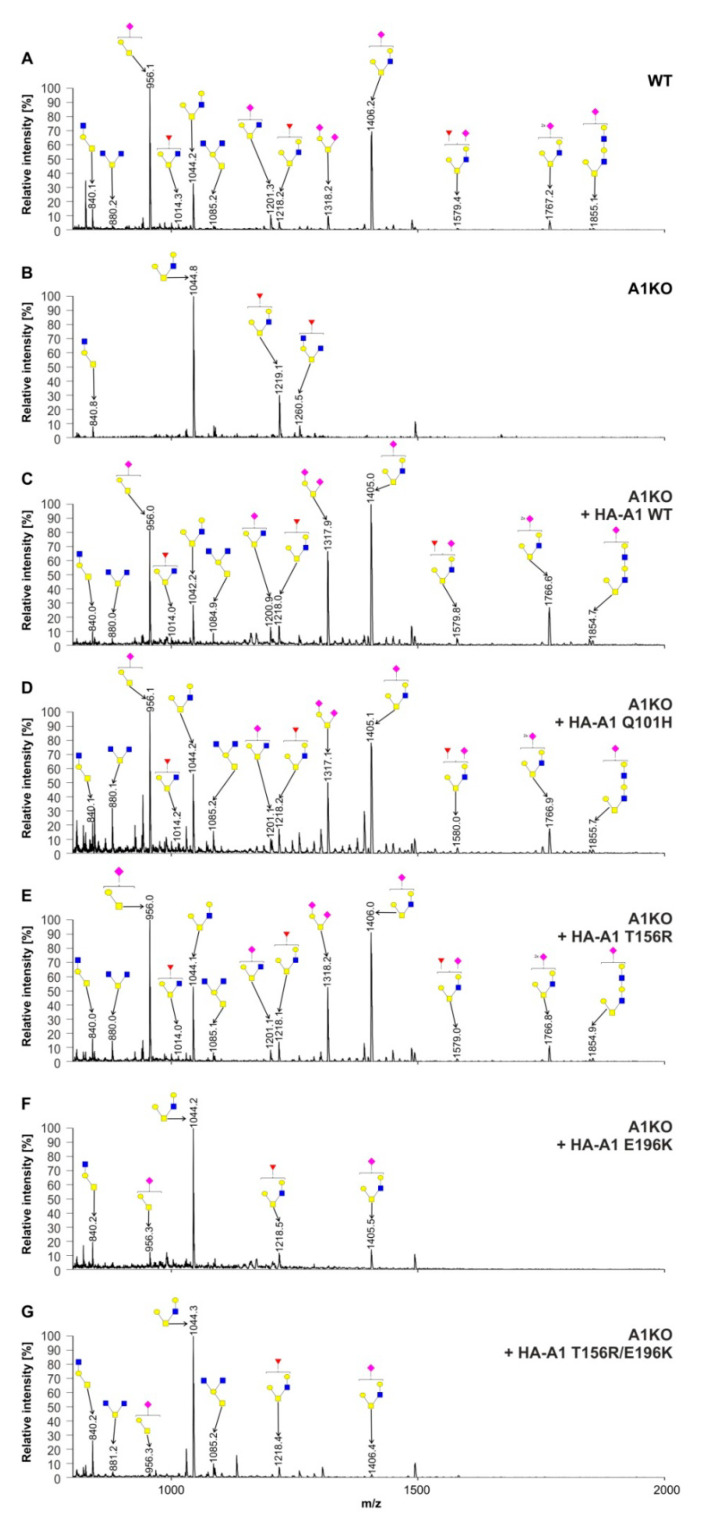
Structural analysis of O-glycans. MALDI-TOF MS characterization of permethylated Bn-O-glycans secreted to culture medium by the wild-type HEK293T cells (WT) (**A**), CST-deficient cells (**B**), CST-deficient cells expressing wild-type CST (**C**), CST-deficient cells expressing the Q101H CST variant (**D**), CST-deficient cells expressing the T156R CST variant (**E**), CST-deficient cells expressing the E196K CST variant (**F**) and CST-deficient cells expressing the T156R/E196K CST variant (**G**). O-Glycan composition was estimated using the GlycoWorkBench tool (2.1; EuroCarbDB). Identified peaks were labeled with mass information and cartoon representations of putative O-glycan chemical structures (based on biosynthetic knowledge). Blue squares, *N*-acetylglucosamine; yellow squares, *N*-acetylgalactosamine; yellow circles, galactose; red triangles, fucose; pink diamonds, sialic acid. A1, SLC35A1.

In wild-type HEK293T cells (Figure 8A) we detected seven sialylated O-glycan species, two of which contained two sialic acid residues (m/z of ≈1318 and ≈1767). In sharp contrast to N-glycans, inactivation of the *SLC35A1* gene resulted in a complete disappearance of all sialylated O-glycan structures (Figure 8B), and in addition, two non-sialylated species (m/z of ≈880 and ≈1085). The knock-out cells synthesized also a fucosylated structure that was not detected in any other of the studied cell lines (m/z of ≈1260). Overexpression of the wild-type (Figure 8C), Q101H (Figure 8D) and T156R (Figure 8E) CST variants triggered the reappearance of all sialylated O-glycans and the two non-sialylated structures that were absent from the profile of the CST-deficient cells. However, the E196K CST variant (Figure 8F) and the double T156R/E196K mutant (Figure 8G) were able to restore only two out of seven sialylated O-glycan species (m/z of ≈956 and ≈1406) and the two non-sialylated structures were only restored by overexpression of the latter.

### 2.7. Analysis of Glycolipids Using Thin-Layer Chromatography

**Figure 9:**
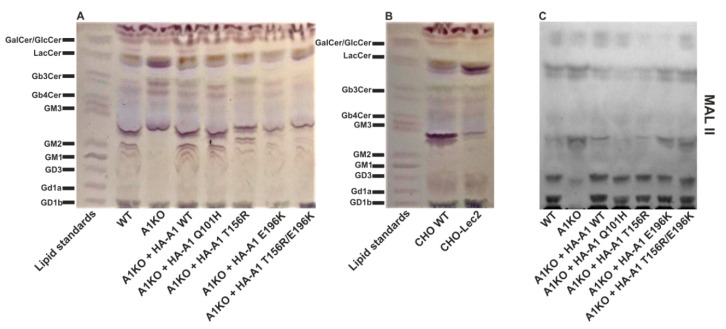
Analysis of glycolipids synthesized by HEK293T (**A**) and CHO (**B**) cell lines using orcinol assay. Total lipids were isolated from cells and separated using thin-layer chromatography, and glycolipids were specifically visualized with the orcinol reagent. A mixture of different standard glycolipids was resolved in parallel. (**C**) Detection of sialylated glycolipids produced by HEK293T cell lines using *Maackia amurensis* lectin II (MAL II). A1, SLC35A1.

Many glycolipids are sialylated. To evaluate the impact of the disease-causing mutations on the sialylated glycolipid species, we extracted total lipids from the cells, separated them by thin-layer chromatography and specifically visualized glycolipids with the orcinol reagent (in the presence of sulfuric acid, neutral carbohydrates present in glycolipids undergo partial dehydration to furfurals, which in turn condense with orcinol upon heating producing violet-brown coloration). As shown in Figure 9A, HEK293T cells synthesize several sialylated glycolipids including Gd1a, GD3, GM1 and GM2. The apparent presence of the two latter species suggests that GM3, which is processed to GM2 in the biosynthetic pathway, should be also produced by these cells. We assume that the band that migrates slightly below the GM3 standard corresponds to this species. The CST-deficient cells did not appear to produce significant amounts of sialylated glycolipids except for the already mentioned GM3, but at the same time displayed a higher subset of neutral glycolipids such as Gb3Cer and LacCer.

Overexpression of the wild-type CST fully restored glycolipid sialylation in the CST-deficient cells, while the mutant CST variants were able to correct defect in sialylation to a various extent. Figure 9B represents glycolipid profiles of CHO and Lec2 cells. In the case of CHO cells sialylated species are barely pronounced except for GM3, which is the sole ganglioside these cells synthesize [58]. We therefore believe that the CST-deficient HEK293T cell line is a better model for studying the relationship between the CST and glycolipid sialylation.

Given the fact that orcinol staining does not discriminate between sialylated and non-sialylated species, we additionally visualized sialylated glycolipids with *Maackia amurensis* lectin II (MAL II). This approach confirmed a signifant reduction in the relative amount of sialylated glycolipids in the knock-out cells (Figure 9C). Again, overexpression of the wild-type CST fully restored glycolipid sialylation but the mutant CST variants were also able to correct this defect.

### 2.8. Analysis of the Ability of CST Variants to Form Dimers Using a Split Luciferase Complementation Assay

**Figure 10:**
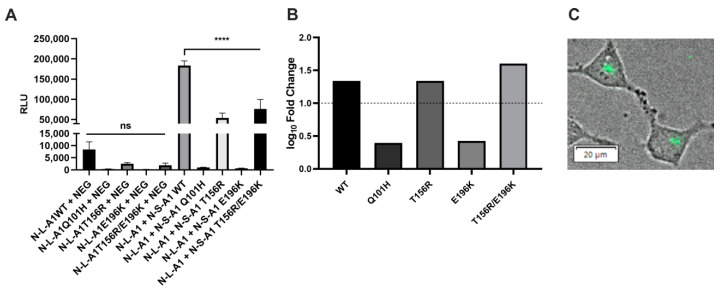
Analysis of dimerization of the CST variants using the NanoBiT approach. (**A**) RLU values obtained for the selected protein combinations and the corresponding negative controls. RLU, relative luminescence units; N-S-, protein tagged with SmBiT at the N-terminus; N-L-, protein tagged with LgBiT at the N-terminus; A1, SLC35A1; NEG, HaloTag tagged with SmBiT. Data were analyzed using one-way ANOVA with multiple comparisons and are presented as a mean ± standard deviation (SD) from three technical replicates. ns, not significant; *p* < 0.001 ****. Representative data from two biological replicates with similar tendencies are shown. (**B**) Decimal logarithms of the ratios calculated by dividing a mean luminescence obtained for the tested combination by a mean luminescence obtained for the corresponding control. The dashed line marks a level of a 10-fold increase (log10 fold change equal 1) of signal over control which sets a threshold of significance suggested by the manufacturer. (**C**) Bioluminescent imaging of the wild-type CST homodimerization in living HEK293T cells. The NanoLuc-derived luminescence (green) was merged with a brightfield image. Concentrated perinuclear localization of the signals strongly suggests that the CST monomers localize to and interact within the Golgi apparatus.

NSTs form homodimers and/or higher homooligomers. The CMP-sialic transporter belongs to the SLC35A subfamily of proteins, and all other members of this subfamily have been reported to homooligomerize [22,23,25,27,59]. Although both mouse and maize CSTs formed homodimers in crystals [41,42] and it was proposed that the dimeric organization of the CST is important for its activity and transport [41], these observations were not supported by any cell-based study. We, therefore, employed the most recent version of a split luciferase complementation assay termed NanoBiT. In this approach, the two proteins of interest are expressed in mammalian cells as fusions with the genetically engineered fragments of the NanoLuc luciferase, SmBiT and LgBiT. Upon the interaction of the fusion proteins, SmBiT and LgBiT reconstitute an active enzyme which generates luminescence after the addition of a cell-permeable substrate. The ability of CST variants to dimerize was analyzed against a control comprising the CST and HaloTag, a protein of bacterial origin not expected to interact with any of mammalian proteins. The luminescence obtained for the wild-type CST was much higher than that of the corresponding control (Figure 10A), and log10 values of the ratios of luminescence values obtained for the tested and control combinations exceeded 1, which is a threshold of significance suggested by the manufacturer (Figure 10B). These findings indicate that the human CST forms dimers in its native environment. We also performed bioluminescent imaging in order to visualize CST dimers within the cells. The NanoLuc-derived signal was concentrated in perinuclear areas (Figure 10C), suggesting that CST dimerization occurs in the Golgi membranes.

Interestingly, all disease-causing CST variants produced significantly lower luminescence than the wild-type variant (Figure 10A). However, when related to the respective controls, the log10 values of the sample:control ratios obtained for the Q101H and E196K variants did not reach the arbitrary threshold of significance, while the log10 values of the sample:control ratios obtained for the T156R variant and the double mutant (T165R + E196K) exceeded the corresponding value (Figure 10B). From these data we concluded that the Q101H and E196K variants, tagged with the NanoBiT fragments and coexpressed in cells, were not able to reconstitute the NanoLuc enzyme to a significant extent. In contrast, wild-type and T156R variants and the double mutant (T165R/E196K) reconstituted NanoLuc to a level exceeding the arbitrary threshold of significance.

## 3. Discussion

In this study we generated and characterized a novel human cell-based model deficient in CMP-sialic acid transport across the Golgi membrane, i.e., HEK293T cells with an inactive *SLC35A1* gene that codes for the CMP-sialic acid transporter. Until recently, the most widely used cell-based model deficient in CST activity was the Lec2 complementation group derived from CHO cell line. However, Lec2 cells were obtained by the means of random mutagenesis, which poses a risk that they might have carried additional mutations outside of the *SLC35A1* gene. Besides, CHO cells are only able to attach sialic acid to galactose residues via α2–3 linkage, as they do not express the gene encoding a sialyltransferase that forms an α2–6 bond [60]. In contrast, HEK293T cells appear to produce the latter enzyme (our unpublished data) and thus should be (and actually are) able to synthesize a greater variety of sialylated glycoconjugates. For example, HEK293T cells synthesize a substantially more diverse repertoire of O-glycans compared to CHO cells [61]. In this work we also demonstrated that HEK293T cells produce a broader range (or higher amounts) of sialylated glycolipids than CHO cells. Finally, while CHO cells are derived from an ovarian tissue, the HEK293T cell line displays some characteristics of neurons [62], which is relevant when taking into account the neurological symptoms experienced by SLC35A1-CDG patients. Altogether, we assumed that the CST-deficient HEK293T cells should be a more appropriate model for studying the functionality of CDG-causing CST variants than the CHO-derived Lec2 mutant cell line. Thus, we inactivated the *SLC35A1* gene in HEK293T cells using a CRISPR/Cas9 strategy. The resulting clone was subsequently used for stable expression of wild-type and selected disease-causing CST variants, giving rise to a panel of seven cell lines that was next subjected to localization studies and in-depth glycophenotypic analyses. We decided to focus on the following mutations: Q101H, described by Mohammed et al. in 2013 [51] and the T156R and E196K mutations occurring in heterozygous configuration, described by Ng et al. in 2017 [54]. We also constructed and characterized an artificial CST variant containing the two latter mutations (T156R/E196K) in a combination.

Congenital disorders of glycosylation are often caused by mislocalization of protein variants that arise due to mutations in the corresponding genes e.g., [63]. Therefore, we first explored the subcellular localization of disease-causing CST variants. The Q101H variant was previously shown to localize to the Golgi apparatus when stably expressed in CHO cells [51]. However, neither T156R nor E196K CST variant was examined in these terms. Here we show that all disease-causing CST variants properly localize to the Golgi apparatus.

In this study we provided a detailed, structural characterization of N- and O-glycans synthesized by HEK293T cells with an inactive *SLC35A1* gene. We also examined glycolipids produced by these cells. The corresponding data represent probably the most comprehensive glycophenotypic analysis of a cell line deficient in CST activity that has ever been performed.

Glycophenotypic analysis of the CST-deficient HEK293T cells revealed only a partial defect in N-glycan sialylation. The obvious persistence of some sialylated N-glycan species in the knock-out cells suggests that residual amounts of CMP-sialic acid are delivered into the Golgi lumen despite the lack of the respective transporter protein. This phenomenon certainly deserves further attention. Lately, Ederveen et al. [64] demonstrated that α2–3 sialylation of serum N-glycans derived from an SLC35A1-CDG patient is affected by the CST deficiency to a more extent than α2–6 sialylation. It is possible that the sialylated N-glycans synthesized by HEK293T cells lacking CST activity contain sialic acid residues attached via α2–6 linkage, although additional analyses are required to confirm this assumption. Nevertheless, in a recently published study [61] we showed that inactivation of the *SLC35A3* gene, which encodes UDP-*N*-acetylglucosamine transporter, does not completely prevent incorporation of *N*-acetylglucosamine into N- and O-glycans by the knock-out cells. Besides, in cells deficient in UDP-galactose transporter activity a residual galactosylation was also revealed [61,65]. It therefore appears that in NST-deficient cells, residual transport of the corresponding nucleotide sugar via an as yet unidentified route(s) is not a marginal effect.

In this work we also examined glycolipids synthesized by the CST-deficient cells. Interestingly, the majority of SLC35A1-CDG patients characterized so far presented with neurological symptoms, which was suggested to be caused by the defects in sialylation of glycoproteins and glycolipids, i.e., gangliosides [51]. The CST impact on glycolipid sialylation has been studied in CHO and Lec2 cells [52,58]. A decrease in the GM3 content accompanied with an increase in the LacCer level have been reported in Lec2 cells. However, as shown by these studies and our results, CHO cells do not synthesize other gangliosides than GM3, whereas HEK293T cells produce a wider range of sialylated glycolipids and are therefore a better model for studying the role that the CST plays in ganglioside biosynthesis than CHO cells. Our data confirm that glycolipid sialylation is significantly impaired in the cells lacking CST activity.

In contrast to N-glycans, sialylation of O-glycans appeared to be completely absent from the knock-out cells. The fact that defects in sialylation of O-glycans were significantly more profound compared to N-glycans strongly suggests that CST deficiency does not evenly affect all classes of glycoconjugates. Our recent work showed that inactivation of the *SLC35A3* gene triggered more significant changes in N-glycans, while O-glycans remained virtually unaltered [61]. It therefore appears that in NST-deficient cells some pathways are better preserved than the others. This may result from different affinities of enzymes involved in distinct glycosylation pathways towards their nucleotide sugar substrates or, alternatively, from diversified ability of mutant NSTs to participate in multi-enzyme, multi-transporter assemblies that were recently demonstrated by Khoder-Agha et al. [27].

Overexpression of disease-causing CST variants in the knock-out cells revealed their uneven potential to rescue sialylation of different classes of glycoconjugates. For instance, the Q101H CST variant did not restore N-glycan sialylation, but appeared fully functional in terms of sialylation of O-glycans. In contrast, the E196K CST variant performed much better in restoring N-glycan sialylation, but in turn barely triggered sialylation of O-glycans. Therefore, for the first time we show that the disease-causing mutations within the *SLC35A1* gene differentially affect distinct classes of glycoconjugates. The ability of individual CST variants to restore sialylation of N- and O-glycans in the knock-out cells is summarized in Table 1.

In our study, the E196K mutation turned out to be more deleterious to CST function than the T156R one. The expression of the E196K variant in the knock-out cells barely restored sialylation of O-glycans and glycolipids in contrast to the T156R variant, whose expression improved sialylation, although less efficiently in the case of N-glycans. Interestingly, although the single T156R and E196K CST variants were both able to moderately improve N-glycan sialylation, their combination turned out to be completely ineffective in these terms. It therefore appears that the combination of these two mutations is more deleterious that one might expect based on functionalities of the single T156R and E196K CST variants.

Interestingly, in the case of CST-deficient cells we observed emergence or accumulation of some species that are not direct precursors of sialylated glycans, e.g., Gb3Cer. The knock-out cells appear to produce GM3, but GM2 and GM1 tend to be decreased, despite the fact that their synthesis from GM3 does not involve any sialylation steps. MALDI-TOF spectra of N- and O-glycans produced by the knock-out cells also revealed some species that are not a direct consequence of undersialylation but suggest alterations at the earlier stages of glycan assembly. This effect could possibly result from undersialylation of the glycosyltransferases that are required for further processing of the discussed species but do not use CMP-sialic acid as a substrate. The majority of glycosyltransferases are glycosylated themselves and it cannot be excluded that some enzymes may not work properly or get mistargeted if the N-glycans they bear are lacking sialic acid.

We also demonstrated the ability of the human CMP-sialic transporter to form dimers in the Golgi membranes of living cells. The Q101H and E196K CST variants, however, did not appear to display such a tendency. The former mutation occurs within the third transmembrane domain (TMD) of CST and was suggested to impair substrate binding [42]. It is thus tempting to speculate whether CST dimerization may be triggered (or at least enhanced) by the binding of CMP-sialic acid to CST monomers. The E196K mutation occurs at the cytosolic side of the sixth TMD of CST and was suggested to impair conformational transitions during transport [42]. Interestingly, the same TMD contains a glycine residue at a position 189 which was shown to be crucial for CST activity [37,66]. Although the T156R CST variant is not expected to dimerize [41], as the mutation occurs in the fifth transmembrane domain which was proposed to mediate dimerization, we did not observe such a behavior, and even more, the double T156R/E196K mutant appeared to form dimers despite the fact that the single E196K mutant failed to reconstitute a functional luciferase in our assay. Unfortunately, at this point we are not able to confirm whether the apparent dimerization of the T156R CST variant corresponds to a physiologically relevant phenomenon. It cannot be excluded that in this case a positive outcome of the NanoBiT assay may correspond to a non-physiological aggregation of CST monomers with a perturbed conformation. Furthermore, a negative result in the NanoBiT approach does not necessarily mean a lack of interaction between the monomers of Q101H and E196K CST variants. We placed the NanoBiT fragments at the N-termini of the studied proteins, as the C-terminus of CST was shown to contain hydrophobic motifs facilitating protein exit from the ER [67]. According to structural data, N-termini of CST monomers are not in a very close proximity within a dimer and do not mediate dimerization. It is therefore possible that Q101H and E196K mutations trigger conformational changes so that the N-termini of the monomers move away from each other, and consequently, a functional luciferase cannot be reconstituted from the fragments even though the proteins they are attached to remain associated. If we, however, assume that Q101H and E196K CST variants are indeed incapable of dimerization, it should be noted that both of them partially corrected sialylation defect in the knock-out cells. This suggests that CST dimerization may not be strictly required for its transporting activity. Interestingly, Hadley et al. [18] performed homology modeling of the CST, based on which they proposed that this protein might be functional both in monomeric and dimeric forms. The results obtained in this study appear to support this assumption.

In conclusion, we generated and extensively characterized a novel human cell line deficient in the CMP-sialic acid transporter activity. Moreover, we determined the ability of CST variants arising as a result of the selected disease-causing mutations to correct the phenotypes of the knock-out cells. Finally, we obtained new data regarding the ability of wild-type and disease-causing CST variants to form dimers in living cells. Our findings extend the knowledge on the CST properties, improve our understanding of the molecular basis of selected SLC35A1-CDG cases and substantiate some of the symptoms the affected individuals tend to experience.

## 4. Materials and Methods

### 4.1. Cell Maintenance

HEK293T cell line was obtained from ATCC, while CHO and Lec2 cell lines were obtained from the Cell Line Collection at the Institute of Immunology and Experimental Therapy, Polish Academy of Sciences, Wroclaw, Poland. Wild-type and knock-out HEK293T cells were grown in DMEM medium supplemented with fetal bovine serum (10%), penicillin (100 U/mL) and streptomycin (100 μg/mL) under standard conditions (37 °C, 5% CO_2_). Knock-out cells overexpressing individual CST variants were cultured in complete DMEM medium with the addition of zeocin (200 μg/mL). CHO and Lec2 cells were grown as described previously [61].

### 4.2. Generation of Knock-Out Cell Lines

HEK293T cell lines lacking a functional *SLC35A1* gene were generated using the CRISPR/Cas9 gene editing approach using guidelines provided by Dharmacon (Lafayette, CO, USA). Briefly, crRNAs targeting three different regions of the *SLC35A1* gene were purchased from Dharmacon. Wild-type cells were co-transfected with the crRNA and tracrRNA complexes and a Cas9-encoding plasmid according to the manufacturer’s instructions. Enrichment for gene-edited cells was done by growing transfected cells in complete DMEM medium supplemented with puromycin (1 µg/mL) for three days. Clones were isolated and primarily screened via labeling of the cell surface with a PNA lectin conjugated with Alexa Fluor-488 (Life Technologies, Carlsbad, CA, USA) followed by analysis using flow cytometry. Gene editing in selected clones was further confirmed using RT-PCR and PCR performed on total cellular RNA and genomic DNA as templates, respectively. The corresponding primers and the expected lengths of the amplified products are listed in Tables S1 and S2.

### 4.3. Generation of Cells Lines Expressing Mutant Variants of the CMP-Sialic Acid Transporter

A cDNA corresponding to the wild-type human *SLC35A1* gene containing a sequence encoding an N-terminal HA tag was amplified using a pTag-GFP2-C-A1 construct [17] and cloned into the pSelect-zeo-mcs plasmid vector (Invivogen, San Diego, CA, USA). Selected mutations were then introduced into the target sequence using the QuikChange Multi Site-Directed Mutagenesis Kit (Agilent Technologies, Santa Clara, CA, USA). Primers used for site-directed mutagenesis and the resulting plasmids are listed in Table S3. The positions of the mutated nucleotides within the *SLC35A1* sequence are shown in Figure S1 and the positions of the corresponding amino acid residues within the CST sequence are shown in Figure S2.

CST-deficient HEK293T cells were transfected with obtained vectors using the FuGENE^®^ HD transfection reagent (Promega, Madison, WI, USA) according to manufacturer’s instructions. Cells were then cultured in DMEM complete medium with the addition of zeocin (400 µg/mL) until stable clones were isolated. Screening for clones stably expressing the desired constructs was performed using immunofluorescence staining with an anti-HA primary antibody followed by a secondary antibody conjugated to an Alexa Fluor dye. Clones were checked for protein expression by Western blotting using HRP-conjugated anti-HA antibody as described previously [65] and PCR carried out on cDNA using gene-specific primers (Tables S1 and S4).

### 4.4. Immunofluorescence Staining

Cells were immunostained as described previously [68]. Primary and secondary antibodies used for immunostaining are listed in Table 2. The resulting samples were analyzed using an LSM510 confocal microscope (Carl Zeiss, Jena, Germany) and the obtained images were processed using ImageJ software 1.48v (NIH, Bethesda, MD, USA).

### 4.5. Lectin Blotting

Cells were lysed, proteins from the resulting lysates were separated using SDS-PAGE, resolved proteins were electrotransferred onto nitrocellulose membranes and they were then probed with biotinylated lectins (Vector Laboratories, Burlingame, CA, USA) listed in Table 3, followed by avidin D conjugated with alkaline phosphatase and colorimetric detection as described previously [65].

### 4.6. Flow Cytometry Analysis of Cells Labeled with Lectins

All sample preparation steps were done on ice. Cells were washed with cold PBS twice and collected in 1 mL of PBS. They were then centrifuged (500× *g*, 5 min, 4 °C), resuspended in 2% BSA in PBS and blocked for 30 min on ice. The cells were then centrifuged again and incubated with lectins, diluted to either 10 µg/mL (PNA conjugated with Alexa Fluor 488, Life Technologies, Carlsbad, CA, USA) or 4 µg/mL (biotinylated MAL II, Vector Laboratories, Burlingame, CA, USA) in 2% BSA in PBS. Unbound lectins were removed by washing the cells twice with 2% BSA in PBS. If a biotinylated lectin was applied, cells were incubated for additional 30 min with streptavidin conjugated with Alexa Fluor 488 (Life Technologies, Carlsbad, CA, USA), diluted 1:100 in 2% BSA in PBS. Finally, the cells were washed twice with 2% BSA in PBS and resuspended in the same solution. Flow cytometry analysis was performed using a NovoCyte Flow Cytometer (ACEA Biosciences, Santa Clara, CA, USA). 10,000 cells were used for each measurement. For statistical analysis of data, one-way ANOVA with the Tukey post-hoc test was used. Analyses were performed with GraphPad Prism (GraphPad Software, CA, USA). Statistical significance was assigned to *p*-value < 0.05.

### 4.7. Preparation of Cell-Derived N-Glycans for HPLC Analysis

Cells were lysed, proteins present in cell lysates were concentrated by acetone precipitation; the resulting samples were subjected to enzymatic deglycosylation; released glycans were isolated, labeled with 2-AB, purified and analyzed as described previously [61].

### 4.8. Preparation of SEAP-Derived N-Glycans for HPLC Analysis

SEAP was transiently overexpressed in the wild-type and CST-deficient cells, purified from the conditioned media and subjected to enzymatic deglycosylation as described previously [61]. Next, isolated N-glycans were labeled with 2-AB, purified and analyzed in the same way as the N-glycans derived from cell lysates.

### 4.9. Exoglycosidase Digestion of SEAP-Derived N-Glycans

For sequential digestion of N-glycans released from SEAP β-*N*-acetylglucosoaminidase S, β1–3 galactosidase, β1–4 galactosidase S and α2–3,6,8,9 neuraminidase A were used (all enzymes were obtained from New England Biolabs, Ipswich, MA, USA). 2-AB-labeled and dried N-glycans were dissolved in 10 µL of 1x GlycoBuffer 1 (New England Biolabs, Ipswich, MA, USA) and incubated overnight at 37 °C with exoglycosidase(s) of interest. After digestion, 3 µL of the reaction mixture was mixed with 7 µL of acetonitrile and directly applied onto the GlycoSepN normal-phase amide column.

### 4.10. Analysis of O-Glycans

To analyze O-glycans, a procedure reported by Kudelka et al. [57] was adopted as described previously [61]. The isolated O-glycans were permethylated and analyzed using MALDI-TOF mass spectrometry in a positive-ion mode.

### 4.11. MALDI-TOF Analysis of N- and O-Glycans

N-Glycans were analyzed using a negative-ion mode. MALDI mass spectra were recorded on an Axima Performance instrument (Shimadzu, Kyoto, Japan) equipped with nitrogen laser (337 nm) in a linear mode. 10 mg of 2,5-dihydroxybenzoic acid in a 10 µL mixture of 1:1 acetonitrile:20 mM ammonium citrate was used as a matrix. All samples were dissolved in 3 µL of 20% acetonitrile. Each mass spectrum was accumulated from at least 200 laser shots and processed using Biotech Launchpad program version 2.9.1 (Shimadzu, Kyoto, Japan). O-Glycans were analyzed in a positive-ion mode as described previously [61].

### 4.12. Analysis of Cellular Glycolipids

Total lipids were extracted from the cells, separated by thin-layer chromatography alongside with the selected glycolipid standards (Biotrend, Köln, Germany) and visualized by orcinol staining as described previously [60]. Thin-layer chromatography of cellular lipids followed by overlaying with biotinylated MAL II, incubation with alkaline phosphatase-conjugated avidin and colorimetric detection was performed as described previously [69].

### 4.13. Split Luciferase Complementation Assay

CST-deficient HEK293T cells were seeded, co-transfected with appropriate expression plasmids and analyzed as described previously [25]. Primers used for amplification of the CST variants tagged with the NanoBiT fragments are listed in Table S5. For statistical analysis of data one-way ANOVA with the Tukey post-hoc test was used. Analyses were performed with GraphPad Prism (GraphPad Software, San Diego, CA, USA). Statistical significance was assigned to *p*-value < 0.05. Only results exceeding the respective negative controls by at least 10-fold were considered indicative of an interaction, as suggested by the manufacturer.

### 4.14. Bioluminescent Imaging

Wild-type HEK293T cells (2 × 10^5^) were seeded in a complete growth medium onto a 35 mm cell culture dish with a glass bottom (Greiner Bio-One, Kremsmünster, Austria). 20–24 h after plating the cells were transfected with a combination of plasmids bearing genes encoding the wild-type CST fused with either large (LgBiT) or small (SmBiT) subunit of NanoLuc luciferase (Table S5, 250 ng of each plasmid, 500 ng in total) using the FuGENE^®^ HD transfection reagent (Promega, Madison, WI, USA); 2–3 h before the measurement the conditioned medium was replaced with the serum-free OPTI-MEM medium (Life Technologies, Carlsbad, CA, USA). Immediately before the measurement, the Live Cell Reagent (Promega, Madison, WI, USA) was added to the cells according to a scaled-up protocol and the cells were immediately examined with an LV200 bioluminescence imaging system (Olympus, Tokyo, Japan).

## Figures and Tables

**Table 1:** The ability of individual CST variants to restore sialylation of N- and O-glycans in the knock-out cells: (-), no rescue; (+), little rescue; (++), moderate rescue; (+++), full rescue.

CST Variant	N-Glycans	O-Glycans
Wild-type	+++	+++
Q101H	-	+++
T156R	++	+++
E196K	++	+
T156R/E196K	-	+

**Table 2:** Antibodies used in immunofluorescence staining experiments.

Antibody	Origin	Dilution	Manufacturer
anti-TGN46	rabbit	1:500	Proteintech (Rosemont, IL, USA)
anti-calnexin	rabbit	1:200	Abcam (Cambridge, UK)
anti-HA	mouse	1:100	Thermo Fisher Scientific (Breda, The Netherlands)
anti-rabbit Alexa Fluor 488	donkey	1:200	Life Technologies (Carlsbad, CA, USA)
anti-mouse Alexa Fluor 568	donkey	1:200	Life Technologies (Carlsbad, CA, USA)

**Table 3:** Lectins used in lectin blotting and dilutions applied.

Lectin	Dilution
PNA	1:200
RCA I	1:200
ECL	1:300

## Data Availability

Data is contained within the article or supplementary material.

## Supplementary Materials

Supplementary materials can be found at https://www.mdpi.com/1422-0067/22/1/304/s1. Figure S1: Nucleotide sequence of a cDNA coding for the wild-type CST. Figure S2: Amino acid sequence of the wild-type CST. Figure S3: Verification of expression of CST variants in stable transfectants on protein (A) and mRNA (B) levels. Table S1: Primers used in RT-PCR analysis of putative *SLC35A1*-deficient clones using total RNA as a template. Table S2: Primers used in PCR analysis of putative *SLC35A1*-deficient clones using genomic DNA as a template. Table S3: Primers used in site-directed mutagenesis. Table S4: Primers used for amplification of the *GAPDH* gene. Table S5: NanoBiT expression plasmids obtained in this study.

## Abbreviations

AB	aminobenzamide
CDG	congenital disorder of glycosylation
CDP	cytidine diphosphate
CHO	Chinese hamster ovary
CMP	cytidine monophosphate
CORA	cellular O-glycome Reporter/amplification
CRISPR	clustered regularly-interspaced short palindromic repeats
CST	CMP-sialic acid transporter
DMEM	Dulbecco’s modified eagle medium
ECL	*Erythrina cristagalli* lectin
ER	endoplasmic reticulum
Gal	galactose
GalNAc	*N*-acetylgalactosamine
GAPDH	glyceraldehyde 3-phosphate dehydrogenase
GDP	guanosine diphosphate
GlcNAc	*N*-acetylglucosamine
GPI	glycosylphosphatidylinositol
HA	hemagglutinin
HEK	human embryonic kidney
HPLC	high performance liquid chromatography
LacCer	lactosylceramide
LgBiT	large NanoLuc subunit
MAG	myelin-associated glycoprotein
MAL/MAA	*Maackia amurensis* lectin/agglutinin
MALDI-TOF	matrix-assisted laser desorption/ionization-time-of-flight
Man	mannose
NanoBiT	NanoLuc Binary Technology
NCAM	neural cell adhesion molecule
Neu5Ac	5-*N*-acetylneuraminic acid
NST	nucleotide sugar transporter
PML	*progressive multifocal leukoencephalopathy*
PNA	*Arachis hypogea* lectin
RCA	*Ricinus communis* agglutinin
SEAP	secreted alkaline phosphatase
SLC	solute carrier
SmBiT	small NanoLuc subunit
TMD	transmembrane domain
UDP	uridine diphosphate
UGT	UDP-galactose transporter
VSV	vesicular stomatitis virus
WGA	wheat germ agglutinin

## References

[1] Varki A. (2008). Sialic acids in human health and disease. Trends Mol. Med..

[2] Fearon D.T. (1978). Regulation by membrane sialic acid of β 1H-dependent decay-dissociation of amplification C3 convertase of the alternative complement pathway. Proc. Natl. Acad. Sci. USA.

[3] Varki A. (2007). Glycan-based interactions involving vertebrate sialic-acid-recognizing proteins. Nature.

[4] Rosen S.D., Singer M., Yednock T., Stoolman L. (1985). Involvement of sialic acid on endothelial cells in organ-specific lymphocyte recirculation. Science.

[5] Moore K.L., Varki A., McEver R.P. (1991). GMP-140 binds to a glycoprotein receptor on human neutrophils: Evidence for a lectin-like interaction. J. Cell Biol..

[6] Rosen S.D. (2004). Ligands for L-selectin: Homing, inflammation, and beyond. Annu. Rev. Immunol..

[7] Collins B.E., Fralich T.J., Itonori S., Ichikawa Y., Schnaar R.L. (2000). Conversion of cellular sialic acid expression from N-acetyl- to N-glycolylneuraminic acid using a synthetic precursor, N-glycolylmannosamine pentaacetate: Inhibition of myelin-associated glycoprotein binding to neural cells. Glycobiology.

[8] Sun J., Shaper N.L., Itonori S., Heffer M., Sheikh K.A., Schnaar R.L. (2004). Myelin-associated glycoprotein (Siglec-4) expression is progressively and selectively decreased in the brains of mice lacking complex gangliosides. Glycobiology.

[9] Varki A., Angata T. (2006). Siglecs—The major subfamily of I-type lectins. Glycobiology.

[10] Schnaar R.L., Gerardy-Schahn R., Hildebrandt H. (2014). Sialic acids in the brain: Gangliosides and polysialic acid in nervous system development, stability, disease, and regeneration. Physiol. Rev..

[11] Sadoul R., Hirn M., Deagostini-Bazin H., Rougon G., Goridis C. (1983). Adult and embryonic mouse neural cell adhesion molecules have different binding properties. Nature.

[12] Rutishauser U., Acheson A., Hall A.K., Mann D.M., Sunshine J. (1988). The neural cell adhesion molecule (NCAM) as a regulator of cell-cell interactions. Science.

[13] Capasso J.M., Hirschberg C.B. (1984). Mechanisms of glycosylation and sulfation in the Golgi apparatus: Evidence for nucleotide sugar/nucleoside monophosphate and nucleotide sulfate/nucleoside monophosphate antiports in the Golgi apparatus membrane. Proc. Natl. Acad. Sci. USA.

[14] Hadley B., Maggioni A., Ashikov A., Day C.J., Haselhorst T., Tiralongo J. (2014). Structure and function of nucleotide sugar transporters: Current progress. Comput. Struct. Biotechnol. J..

[15] Chintala S., Tan J., Gautam R., Rusiniak M.E., Guo X., Li W., Gahl W.A., Huizing M., Spritz R.A., Hutton S. (2007). The Slc35d3 gene, encoding an orphan nucleotide sugar transporter, regulates platelet-dense granules. Blood.

[16] Wei Z., Yuan Y.-F., Jaouen F., Ma M.-S., Hao C.-J., Zhang Z., Chen Q., Yuan Z., Yu L., Li W. (2016). SLC35D3 increases autophagic activity in midbrain dopaminergic neurons by enhancing BECN1-ATG14-PIK3C3 complex formation. Autophagy.

[17] Sosicka P., Maszczak-Seneczko D., Bazan B., Shauchuk Y., Kaczmarek B., Olczak M. (2017). An insight into the orphan nucleotide sugar transporter SLC35A4. Biochim. Biophys. Acta Mol. Cell Res..

[18] Hadley B., Litfin T., Day C.J., Haselhorst T., Zhou Y., Tiralongo J. (2019). Nucleotide sugar transporter SLC35 family structure and function. Comput. Struct. Biotechnol. J..

[19] Puglielli L., Hirschberg C.B. (1999). Reconstitution, identification, and purification of the rat liver Golgi membrane GDP-fucose transporter. J. Biol. Chem..

[20] Puglielli L., Mandon E.C., Rancour D.M., Menon A.K., Hirschberg C.B. (1999). Identification and purification of the rat liver Golgi membrane UDP-N-acetylgalactosamine transporter. J. Biol. Chem..

[21] Gao X.-D., Dean N. (2000). Distinct protein domains of the yeast Golgi GDP-mannose transporter mediate oligomer assembly and export from the endoplasmic reticulum. J. Biol. Chem..

[22] Olczak M., Guillen E. (2006). Characterization of a mutation and an alternative splicing of UDP-galactose transporter in MDCK-RCA^r^ cell line. Biochim. Biophys. Acta.

[23] Maszczak-Seneczko D., Sosicka P., Majkowski M., Olczak T., Olczak M. (2012). UDP-N-acetylglucosamine transporter and UDP-galactose transporter form heterologous complexes in the Golgi membrane. FEBS Lett..

[24] Parker J.L., Newstead S. (2017). Structural basis of nucleotide sugar transport across the Golgi membrane. Nature.

[25] Wiertelak W., Sosicka P., Olczak M., Maszczak-Seneczko D. (2020). Analysis of homologous and heterologous interactions between UDP-galactose transporter and beta-1,4-galactosyltransferase 1 using NanoBiT. Anal. Biochem..

[26] Maszczak-Seneczko D., Sosicka P., Kaczmarek B., Majkowski M., Luzarowski M., Olczak T., Olczak M. (2015). UDP-galactose (SLC35A2) and UDP-N-acetylglucosamine (SLC35A3) transporters form glycosylation-related complexes with mannoside acetylglucosaminyltransferases (Mgats). J. Biol. Chem..

[27] Khoder-Agha F., Sosicka P., Conde M.E., Hassinen A., Glumoff T., Olczak M., Kellokumpu S. (2019). N-acetylglucosaminyltransferases and nucleotide sugar transporters form multi-enzyme–multi-transporter assemblies in golgi membranes in vivo. Cell. Mol. Life Sci..

[28] Shauchuk A., Szulc B., Maszczak-Seneczko D., Wiertelak W., Skurska E., Olczak M. (2020). N-glycosylation of the human β1,4-galactosyltransferase 4 is crucial for its activity and Golgi localization. Glycoconj. J..

[29] Kellokumpu S., Hassinen A., Glumoff T. (2015). Glycosyltransferase complexes in eukaryotes: Long-known, prevalent but still unrecognized. Cell. Mol. Life Sci..

[30] Stanley P., Siminovitch L. (1977). Complementation between mutants of CHO cells resistant to a variety of plant lectins. Somat. Cell Mol. Genet..

[31] Briles E.B., Li E., Kornfeld S. (1977). Isolation of wheat germ agglutinin-resistant clones of Chinese hamster ovary cells deficient in membrane sialic acid and galactose. J. Biol. Chem..

[32] Deutscher S.L., Nuwayhid N., Stanley P., Briles E.I.B., Hirschberg C.B. (1984). Translocation across golgi vesicle membranes: A CHO glycosylation mutant deficient in CMP-sialic acid transport. Cell.

[33] Eckhardt M., Mühlenhoff M., Bethe A., Gerardy-Schahn R. (1996). Expression cloning of the Golgi CMP-sialic acid transporter. Proc. Natl. Acad. Sci. USA.

[34] Eckhardt M., Gerardy-Schahn R. (1997). Molecular cloning of the hamster CMP-sialic acid transporter. Eur. J. Biochem..

[35] Berninsone P., Eckhardt M., Gerardy-Schahn R., Hirschberg C.B. (1997). Functional expression of the murine Golgi CMP-sialic acid transporter in Saccharomyces cerevisiae. J. Biol. Chem..

[36] Ishida N., Ito M., Yoshioka S., Sun-Wada G.-H., Kawakita M. (1998). Functional expression of human golgi CMP-sialic acid transporter in the Golgi complex of a transporter-deficient Chinese hamster ovary cell mutant. J. Biochem..

[37] Lim S.F., Lee M.M., Zhang P., Song Z. (2008). The Golgi CMP-sialic acid transporter: A new CHO mutant provides functional insights [published correction appears in *Glycobiology*
**2009**, *19*, 192]. Glycobiology.

[38] Eckhardt M., Gotza B., Gerardy-Schahn R. (1999). Membrane topology of the mammalian CMP-sialic acid transporter. J. Biol. Chem..

[39] Aoki K., Sun-Wada G.-H., Segawa H., Yoshioka S., Ishida N., Kawakita M. (1999). Expression and activity of chimeric molecules between human UDP-galactose transporter and CMP-sialic acid transporter. J. Biochem..

[40] Aoki K., Ishida N., Kawakita M. (2001). Substrate recognition by UDP-galactose and CMP-sialic acid transporters. Different sets of transmembrane helices are utilized for the specific recognition of UDP-galactose and CMP-sialic acid. J. Biol. Chem..

[41] Nji E., Gulati A., Qureshi A.A., Coincon M., Drew D. (2019). Structural basis for the delivery of activated sialic acid into Golgi for sialyation. Nat. Struct. Mol. Biol..

[42] Ahuja S., Whorton M.R. (2019). Structural basis for mammalian nucleotide sugar transport. Elife.

[43] Parker J.L., Corey R.A., Stansfeld P.J., Newstead S. (2019). Structural basis for substrate specificity and regulation of nucleotide sugar transporters in the lipid bilayer. Nat. Commun..

[44] Ströh L.J., Stehle T. (2014). Glycan engagement by viruses: Receptor switches and specificity. Annu. Rev. Virol..

[45] Urbanek K., Sutherland D.M., Orchard R.C., Wilen C.B., Knowlton J.J., Aravamudhan P., Taylor G.M., Virgin H.W., Dermody T.S. (2020). Cytidine monophosphate N-acetylneuraminic acid synthetase and solute carrier family 35 member A1 are required for reovirus binding and infection. J. Virol..

[46] Han J., Perez J.T., Chen C., Li Y., Benitez A., Kandasamy M., Lee Y., Andrade J., Tenoever B., Manicassamy B. (2018). Genome-wide CRISPR/Cas9 screen identifies host factors essential for influenza virus replication. Cell Rep..

[47] Mascarenhas J.X., Korokhov N., Burger L., Kassim A., Tuter J., Miller D., Borgschulte T., George H.J., Chang A., Pintel D.J. (2017). Genetic engineering of CHO cells for viral resistance to minute virus of mice. Biotechnol. Bioeng..

[48] Moskovskich A., Goldmann U., Kartnig F., Lindinger S., Konecka J., Fiume G., Girardi E., Superti-Furga G. (2019). The transporters SLC35A1 and SLC30A1 play opposite roles in cell survival upon VSV virus infection. Sci. Rep..

[49] Geoghegan E.M., Pastrana D.V., Schowalter R.M., Ray U., Gao W., Ho M., Pauly G.T., Sigano D.M., Kaynor C., Cahir-McFarland E. (2017). Infectious entry and neutralization of pathogenic JC polyomaviruses. Cell Rep..

[50] Martínez-Duncker I., Dupré T., Piller V., Piller F., Candelier J.-J., Trichet C., Tchernia G., Oriol R., Mollicone R. (2005). Genetic complementation reveals a novel human congenital disorder of glycosylation of type II, due to inactivation of the Golgi CMP-sialic acid transporter. Blood.

[51] Mohamed M., Ashikov A., Guillard M., Robben J.H., Schmidt S., Heuvel B.V.D., De Brouwer A.P., Gerardy-Schahn R., Deen P.M., Wevers R.A. (2013). Intellectual disability and bleeding diathesis due to deficient CMP-sialic acid transport. Neurology.

[52] Riemersma M., Sandrock J., Boltje T.J., Büll C., Heise T., Ashikov A., Adema G.J., van Bokhoven H., Lefeber D.J. (2015). Disease mutations in CMP-sialic acid transporter SLC35A1 result in ab-normal alpha-dystroglycan O-mannosylation, independent from sialic acid. Hum. Mol. Genet..

[53] Van Tol W., Alsady M., van Hove H., van Scherpenzeel M., Willemsen M.A., Ashikov A., Lefeber D.J. (2020). Ribitol Supplementation Restores the O-Mannosyl Glycans of Alpha-Dystroglycan in SLC35A1 Deficiency (Chapter 5). Ph.D. Thesis.

[54] Ng B.G., Asteggiano C.G., Kircher M., Buckingham K.J., Raymond K., Nickerson D.A., Shendure J., Bamshad M.J., Ensslen M., Freeze H.H. (2017). Encephalopathy caused by novel mutations in the CMP-sialic acid transporter, SLC35A1. Am. J. Med. Genet. A.

[55] Kauskot A., Pascreau T., Adam F., Bruneel A., Reperant C., Lourenco-Rodrigues M.-D., Rosa J.-P., Petermann R., Maurey H., Auditeau C. (2018). A mutation in the gene coding for the sialic acid transporter SLC35A1 is required for platelet life span but not proplatelet formation. Haematologica.

[56] Ma X., Li Y., Kondo Y., Shi H., Han J., Jiang Y., Bai X., Archer-Hartmann S.A., Azadi P., Ruan C. (2020). Slc35a1 deficiency causes thrombocytopenia due to impaired megakaryocytopoiesis and excessive platelet clearance in the liver. Haematologica.

[57] Kudelka M.R., Antonopoulos A., Wang Y., Duong D.M., Song X., Seyfried N.T., Dell A., Haslam S.M., Cummings R.D., Ju T. (2016). Cellular O-glycome reporter/amplification to explore O-glycans of living cells. Nat. Methods.

[58] Stanley P., Sudo T., Carver J.P. (1980). Differential involvement of cell surface sialic acid residues in wheat germ agglutinin binding to parental and wheat germ agglutinin-resistant Chinese hamster ovary cells. J. Cell Biol..

[59] Sosicka P., Bazan B., Maszczak-Seneczko D., Shauchuk Y., Olczak T., Olczak M. (2019). SLC35A5 Protein—A Golgi complex member with putative nucleotide sugar transport activity. Int. J. Mol. Sci..

[60] Lee E.U., Roth J., Paulson J.C. (1989). Alteration of terminal glycosylation sequences on *N*-Linked oligosaccharides of Chinese hamster ovary cells by expression of β-galactoside α2,6-sialyltransferase. J. Biol. Chem..

[61] Szulc B., Sosicka P., Maszczak-Seneczko D., Skurska E., Shauchuk A., Olczak T., Freeze H.H., Olczak M. (2020). Biosynthesis of GlcNAc-rich N- and O-glycans in the Golgi apparatus does not require the nucleotide sugar transporter SLC35A3. J. Biol. Chem..

[62] Shaw G., Morse S., Ararat M., Graham F.L. (2002). Preferential transformation of human neuronal cells by human adenoviruses and the origin of HEK 293 cells. FASEB J..

[63] Marquardt T., Bzduch V., Hogrebe M., Rust S., Reunert J., Grüneberg M., Park J., Callewaert N., Lachmann R., Wada Y. (2020). SLC37A4-CDG: Mislocalization of the glucose-6-phosphate transporter to the Golgi causes a new congenital disorder of glycosylation. Mol. Genet. Metab. Rep..

[64] Ederveen A.L.H., de Haan N., Baerenfaenger M., Lefeber D.J., Wuhrer M. (2020). Dissecting total plasma and protein-specific glycosylation profiles in congenital disorders of glycosylation. Int. J. Mol. Sci..

[65] Maszczak-Seneczko D., Olczak T., Wunderlich L., Olczak M. (2011). Comparative analysis of involvement of UGT1 and UGT2 splice variants of UDP-galactose transporter in glycosylation of macromolecules in MDCK and CHO cell lines. Glycoconj. J..

[66] Eckhardt M., Gotza B., Gerardy-Schahn R. (1998). Mutants of the CMP-sialic acid transporter causing the Lec2 phenotype. J. Biol. Chem..

[67] Zhao W., Chen T.-L.L., Vertel B.M., Colley K.J. (2006). The CMP-sialic acid transporter is localized in the medial-trans Golgi and possesses two specific endoplasmic reticulum export motifs in its carboxyl-terminal cytoplasmic tail. J. Biol. Chem..

[68] Bazan B., Wiktor M., Maszczak-Seneczko D., Olczak T., Kaczmarek B., Olczak M. (2018). Lysine at position 329 within a C-terminal dilysine motif is crucial for the ER localization of human SLC35B4. PLoS ONE.

[69] Kaczmarek R., Mikolajewicz K., Szymczak K., Duk M., Majorczyk E., Krop-Watorek A., Buczkowska A., Czerwinski M. (2016). Evaluation of an amino acid residue critical for the specificity and activity of human Gb3/CD77 synthase. Glycoconj. J..

