# Supplementary material for: Novel Insights into Selected Disease-Causing Mutations within the SLC35A1 Gene Encoding the CMP-Sialic Acid Transporter
Source: Int J Mol Sci. 2020 Dec 30;22(1):304. doi: 10.3390/ijms22010304 (PMC7795627; doi:10.3390/ijms22010304)
Supplement: Supplementary file 1 [file ijms-22-00304-s001.pdf]

```
1    ATGGCTGCCCCGAGAGACAATGTCACTTTATTATTCAAGTTATACTGCTTGGCAGTGATGACCCTGATGGCTGCAGTCTATACCATAGCTTTAAGATACA
101  CAAGGACATCAGACAAAGAACTCTACTTTTCAACCACAGCCGTGTGTATCACAGAAGTTATAAAGTTATTGCTAAGTGTGGGAATTTTAGCTAAAGAAAC
201  TGGTAGTCTGGGTAGATTCAAAGCATCTTTAAGAGAAAATGTCTTGGGGAGCCCCAAGGAACTGTTGAAGTTAAGTGTGCCATCGTTAGTGTATGCTGTT
301  CAGAACAACATGGCTTTCCTAGCTCTTAGCAATCTGGATGCAGCAGTGTACCAGGTGACCTACCAGTTGAAGATTCCGTGTACTGCTTTATGCACTGTTT
401  TAATGTTAAACCGGACACTCAGCAAATTACAGTGGGTTTCAGTTTTTATGCTGTGTGCTGGAGTTACGCTTGTACAGTGGAAACCAGCCCAAGCTACAAA
501  AGTGGTGGTGGAACAAAATCCATTATTAGGGTTTGGCGCTATAGCTATTGCTGTATTGTGCTCAGGATTTGCAGGAGTATATTTTGAAAAAGTTTTAAAG
601  AGTTCAGATACTTCTCTTTGGGTGAGAAACATTCAAATGTATCTATCAGGGATTATTGTGACATTAGCTGGCGTCTACTTGTCAGATGGAGCTGAAATTA
701  AAGAAAAAGGATTTTTCTATGGTTACACATATTATGTCTGGTTTGTCATCTTTCTTGCAAGTGTTGGTGGCCTCTACACTTCTGTTGTGGTTAAGTACAC
801  AGACAACATCATGAAAGGCTTTTCTGCAGCAGCGGCCATTGTCCTTTCCACCATTGCTTCAGTAATGCTGTTTGGATTACAGATAACACTCACCTTTGCC
901  CTGGGTACTCTTCTTGTATGTGTTTCCATATATCTCTATGGATTACCCAGACAAGACACTACATCCATCCAACAAGGAGAAACAGCTTCAAAGGAGAGAG
1001 TTATTGGTGTGTGA
```

Figure S1. Nucleotide sequence of a cDNA coding for the wild-type CST. Nucleotides that were subjected to mutations are labeled in the following colors: light-blue (c.303 G>C), purple (c.467 C>G) and dark-blue (c.586 G>A).

```
1    MAAPRDNVTLLFKLYCLAVMTLMAAVYTIALRYTRTSDKELYFSTTAVCI
51   TEVIKLLLSVGILAKETGSLGRFKASLRENVLGSPKELLKLSVPSLVYAV
101  QNNMAFLALSNLDAAVYQVTYQLKIPCTALCTVLMLNRTLSKLQWVSVFM
151  LCAGVTLVQWKPAQATKVVVEQNPLLGFGAIAIAVLCSGFAGVYFEKVLK
201  SSDTSLWVRNIQMYLSGIIVTLAGVYLSDGAEIKEKGFFYGYTYYVWFVI
251  FLASVGGLYTSVVVKYTDNIMKGFSAAAAIVLSTIASVMLFGLQITLTFA
301  LGTLLVCVSIYLYGLPRQDTTSIQQGETASKERVIGV
```

Figure S2. Amino acid sequence of the wild-type CST. Amino acids that were subjected to mutations are labeled in the following colors: light-blue (p.Q101H), purple (p.T156R) and dark-blue (p.E196K).

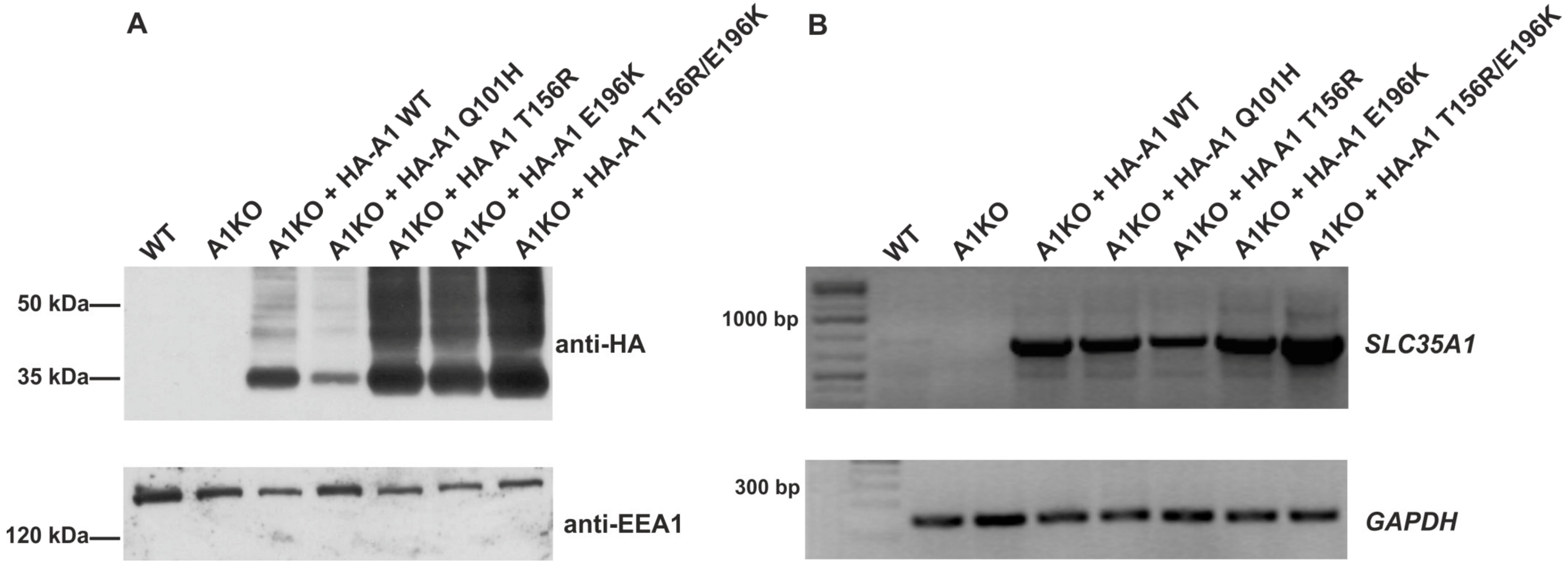

Figure S3. Verification of expression of CST variants in stable transfectants on protein (A) and mRNA (B) levels. (A) Whole cell lysates were separated by SDS-PAGE, resolved proteins were electrotransferred onto nitrocellulose membrane and HA-tagged CST variants were detected by Western blotting using HRP-conjugated anti-HA antibody followed by chemiluminescent detection. Early endosome antigen 1 (EEA1) was detected in parallel as a loading control. (B) Total RNA was isolated from cells, cDNA was synthesized from the mRNA template and semi-quantitative PCR was carried out using primers designed to amplify the *SLC35A1* gene (Table S1). The *GAPDH* gene was amplified in parallel as a reference using primers listed in Table S4.

Table S1. Primers used in RT-PCR analysis of putative *SLC35A1*-deficient clones using total RNA as a template.

Primer name	Primer sequence	Product length
F1 crRNA 1&3	ACTGCTTGGCAGTGATGACC	927 bp
R2 crRNA 2	GGATGGATGTAGTGTCTTGTCTGG	

Table S2. Primers used in PCR analysis of putative *SLC35A1*-deficient clones using genomic DNA as a template.

Primer name	Primer sequence	Product length	Product localization
F1 crRNA 1&3	ACTGCTTGGCAGTGATGACC	351 bp	Exon 2
R2i crRNA 1&3	CCTTGGTCTCCACCCACTAG		
F2i crRNA 2	GTGGTCAGATAGTGTCAGTTAGGC	365 bp	Exon 8
R1 crRNA 2	GCTGTTTCTCCTTGTTGGATGG		

Table S3. Primers used in site-directed mutagenesis. Mutated nucleotides are indicated in red.

Primer name	Primer sequence	Resulting construct	Mutated nucleotide	Mutated amino acid
F_A1_303	CGTTAGTGTATGCTGTTCACAACAACATGGCTTTCCTAGC	pSelect-HA-SLC35A1(303)	c.303 G>C	p.Q101H
F_A1_467	TATGCTGTGTGCTGGAGTTAGGCTTGTACAGTGG	pSelect-HA-SLC35A1(467)	c.467 C>G	p.T156R
F_A1_586	GTGCTCAGGATTTGCAGGAGTATATTTTAAAAAAGTTTTAAAGAGTTCAG	pSelect-HA-SLC35A1(586)	c.586 G>A	p.E196K

Table S4. Primers used for amplification of the *GAPDH* gene.

Primer name	Primer sequence	Product length
Forward	AGGTCGGAGTCAACGGATTT	192 bp
Reverse	TGACAAGCTTCCCGTTCTCA	

Table S5. NanoBiT expression plasmids obtained in this study.

Construct	Forward primer	Reverse primer	Backbone plasmid
N-L-A1 WT	AAAAGAGCTCAGATGGCTGCCCCGAGAG	AAAAGAATTCTCACACACCAATAACTCTCTCCTTTGA	pBiT1.1-N[TK/LgBiT]
N-S-A1 WT	AAAAGAGCTCAGATGGCTGCCCCGAGAG	AAAAGAATTCTCACACACCAATAACTCTCTCCTTTGA	pBiT2.1-N[TK/SmBiT]
N-L-A1 Q101H	AAAAGAGCTCAGATGGCTGCCCCGAGAG	AAAAGAATTCTCACACACCAATAACTCTCTCCTTTGA	pBiT1.1-N[TK/LgBiT]
N-S-A1 Q101H	AAAAGAGCTCAGATGGCTGCCCCGAGAG	AAAAGAATTCTCACACACCAATAACTCTCTCCTTTGA	pBiT2.1-N[TK/SmBiT]
N-L-A1 T156R	AAAAGAGCTCAGATGGCTGCCCCGAGAG	AAAAGAATTCTCACACACCAATAACTCTCTCCTTTGA	pBiT1.1-N[TK/LgBiT]
N-S-A1 T156R	AAAAGAGCTCAGATGGCTGCCCCGAGAG	AAAAGAATTCTCACACACCAATAACTCTCTCCTTTGA	pBiT2.1-N[TK/SmBiT]
N-L-A1 E196K	AAAAGAGCTCAGATGGCTGCCCCGAGAG	AAAAGAATTCTCACACACCAATAACTCTCTCCTTTGA	pBiT1.1-N[TK/LgBiT]
N-S-A1 E196K	AAAAGAGCTCAGATGGCTGCCCCGAGAG	AAAAGAATTCTCACACACCAATAACTCTCTCCTTTGA	pBiT2.1-N[TK/SmBiT]
N-L-A1 T156R/E196K	AAAAGAGCTCAGATGGCTGCCCCGAGAG	AAAAGAATTCTCACACACCAATAACTCTCTCCTTTGA	pBiT1.1-N[TK/LgBiT]
N-S-A1 T156R/E196K	AAAAGAGCTCAGATGGCTGCCCCGAGAG	AAAAGAATTCTCACACACCAATAACTCTCTCCTTTGA	pBiT2.1-N[TK/SmBiT]
